# *In Vitro* and *In Vivo* Prostate Cancer Metastasis and Chemoresistance Can Be Modulated by Expression of either CD44 or CD147

**DOI:** 10.1371/journal.pone.0040716

**Published:** 2012-08-03

**Authors:** Jingli Hao, Michele C. Madigan, Aparajita Khatri, Carl A. Power, Tzong-Tyng Hung, Julia Beretov, Lei Chang, Weiwei Xiao, Paul J. Cozzi, Peter H. Graham, John H. Kearsley, Yong Li

**Affiliations:** 1 St George Clinical School, University of New South Wales (UNSW), Kensington, New South Wales, Australia; 2 Cancer Care Centre, St George Hospital, Kogarah, New South Wales, Australia; 3 School of Optometry and Vision Science, University of New South Wales (UNSW), Kensington, New South Wales, Australia, and Save Sight Institute, Sydney Medical School, University of Sydney, Sydney, New South Wales, Australia; 4 Prince of Wales Clinical School, University of New South Wales (UNSW), Kensington, New South Wales, Australia; 5 Biological Resources Imaging Laboratory, University of New South Wales (UNSW), Kensington, New South Wales, Australia; 6 Department of Anatomical Pathology, St George Hospital, Kogarah, New South Wales, Australia; 7 Department of Surgery, St George Hospital, Kogarah, New South Wales, Australia; The University of Texas M.D. Anderson Cancer Center, United States of America

## Abstract

CD44 and CD147 are associated with cancer metastasis and progression. Our purpose in the study was to investigate the effects of down-regulation of CD44 or CD147 on the metastatic ability of prostate cancer (CaP) cells, their docetaxel (DTX) responsiveness and potential mechanisms involved *in vitro* and *in vivo*. CD44 and CD147 were knocked down (KD) in PC-3M-luc CaP cells using short hairpin RNA (shRNA). Expression of CD44, CD147, MRP2 (multi-drug resistance protein-2) and MCT4 (monocarboxylate tranporter-4) was evaluated using immunofluorescence and Western blotting. The DTX dose-response and proliferation was measured by MTT and colony assays, respectively. The invasive potential was assessed using a matrigel chamber assay. Signal transduction proteins in PI3K/Akt and MAPK/Erk pathways were assessed by Western blotting. An *in vivo* subcutaneous (s.c.) xenograft model was established to assess CaP tumorigenecity, lymph node metastases and DTX response. Our results indicated that KD of CD44 or CD147 decreased MCT4 and MRP2 expression, reduced CaP proliferation and invasive potential and enhanced DTX sensitivity; and KD of CD44 or CD147 down-regulated p-Akt and p-Erk, the main signal modulators associated with cell growth and survival. *In vivo*, CD44 or CD147-KD PC-3M-luc xenografts displayed suppressed tumor growth with increased DTX responsiveness compared to control xenografts. Both CD44 and CD147 enhance metastatic capacity and chemoresistance of CaP cells, potentially mediated by activation of the PI3K and MAPK pathways. Selective targeting of CD44/CD147 alone or combined with DTX may limit CaP metastasis and increase chemosensitivity, with promise for future CaP treatment.

## Introduction

Prostate cancer (CaP) is the most commonly diagnosed and the second leading cause of death from cancer in males in the USA [1]. While initially responsive to androgen deprivation therapy (ADT), most patients suffer from cancer recurrence after 12 months, and CaP at this stage is no longer curable [2]. The progression of CaP to an advanced stage is characterized by the dissemination of malignant cancer cells and small cell clusters through lymphatics and blood vessels. Although several genetic and epigenetic changes are reported in CaP progression, the cellular mechanisms involved in progression of localized CaP to metastatic disease remain undefined.

Chemotherapy is traditionally used for palliation of symptoms associated with advanced CaP. Two recent docetaxel (DTX)-based clinical studies have for the first time shown the potential benefits of chemotherapy to prolong the survival time and life quality of CaP patients [3], [4]. DTX is currently the most effective chemotherapeutic drug for metastatic CaP [3]–[6]. However, the drug-resistant nature of CaP still challenges the effectiveness of such therapies. Clearly, multidrug resistance and metastatic disease remain the main causes of treatment failure and mortality in CaP patients. Thus, it is of significant value to investigate the mechanisms and pathways of CaP metastasis and drug resistance, identify useful therapeutic targets to improve current therapeutic modalities.

CD44 is a multifunctional protein involved in cell adhesion, migration, drug resistance, signal transmission, migration and apoptosis [7], [8]. The CD44 gene contains 20 exons alternatively spliced to give many isoforms or variants (CD44v), some of which form the invariant extracellular domain of standard CD44 (CD44s). CD44 is a primary receptor for hyaluronan (HA), a major component of the extracellular matrix (ECM) and critical for cell signaling and cell-ECM interactions in cancer. CD44-HA binding stimulates downstream effects on cytoskeleton proteins implicated in tumor cell migration, as well as stimulating multidrug resistance protein1 (*MDR1*) expression and drug resistance [9]. CD44s is present on the membrane of most vertebrate cells [7]. The expression of certain CD44 variants is reported to be closely associated with tumor progression, and this varies depending on tumor type studied [10]. Intriguing studies have implicated HA/CD44 interactions in epithelial mesenchymal-transition (EMT), “stemness” and cancer [11], [12]. The interaction between HA and CD44 has been shown to trigger pathways related to tumor growth and survival [13], [14]. However, the role of CD44s and CD44v in CaP development and progression is different, with studies showing both tumor-inhibiting (CD44s) and tumor-promoting (CD44v) effects [15]–[17]. The involvement of CD44 and its variants in CaP progression and metastases warrants further investigation.

CD147 (extracellular matrix metalloproteinase inducer protein-EMMPRIN) is a multifunctional glycoprotein that can modify tumor microenvironment by activating proteinases, inducing angiogenic factors in both tumor and stromal cells, and regulating growth and survival of anchorage-independent tumor cells (micrometastases) as well as multidrug resistance [18]. Transcriptome analysis and comparative genomic hybridization of individual tumor cells isolated from bone marrow of patients with CaP showed that CD147 is the most frequently expressed protein in primary tumors and micrometastases [19]. Moreover, increased CD147 expression is associated with increased risks including prostate specific antigen (PSA) failure, metastasis, and reduced overall survival in human CaP [20]. We recently showed that the high levels of CD147 expression correlate with CaP progression and are associated with the expression of MMPs (matrix metalloproteinases) in tumor as well as stromal cells [21]. These results suggest that CD147 could be a useful therapeutic target for CaP therapy. However, the role of CD147 in CaP metastasis and drug resistance remains unclear.

In the current study, we hypothesized that (1) CD44 and CD147 expression correlate with the metastatic capacity and chemoresistance of CaP and may co-operate to influence CaP progression and (2) down-regulation of CD44 or CD147 expression could have therapeutic potential in limiting CaP metastasis and enhancing CaP chemotherapeutic sensitivity. We demonstrate in the following sections that CD44 and CD147 confer properties significant for CaP metastasis and chemoresistance *in vitro* and *in vivo*, and are useful therapeutic targets for future CaP therapy.

## Materials and Methods

### Antibodies

Antibodies were obtained from different sources. The detailed information and conditions for all antibodies are listed in **Table** **1**.

**Table 1:** Antibodies used for western blot (WB), immunofluorescence (IF), immunohistochemistry (IHC) staining and immunoprecipitation (IP).

Antibody	Source	Type	Dilution	Incubation time (min)	Temperature	Application
Mouse anti-human CD44 (DF1485)	Santa Cruz Biotechnology	MAb	1∶200 (WB, IF)	O/N	4°C	WB, IF, IP
Rabbit anti-human CD147	Invitrogen	PAb	1∶400 (WB) 1∶200 (IF)	O/N	4°C	WB, IF, IP
Rabbit anti-human MCT1	Santa Cruz Biotechnology	PAb	1∶400 (WB, IF)	O/N	4°C	WB, IF
Rabbit anti-human MCT4	Santa Cruz Biotechnology	PAb	1∶400 (WB, IF)	O/N	4°C	WB, IF
Mouse anti-human MRP2	Enzo Life Sciences	PAb	1∶100 (WB) 1∶50 (IF)	O/N	4°C	WB, IF
Rabbit anti-human phospho-Merlin (Ser518) antibody	Cell Signaling Technology	PAb	1∶1000 (WB)	O/N	4°C	WB
Rabbit anti-human Akt1/2/3 (H-136)	Santa Cruz Biotechnology	PAb	1∶200	O/N	4°C	WB
Rabbit anti-human Phospho-Akt	Cell Signaling Technology	PAb	1∶500 (WB)	O/N	4°C	WB
Rabbit anti-human p44/42 MAPK (Erk1/2)	Cell Signaling Technology	PAb	1∶500 (WB)	O/N	4°C	WB
Rabbit anti-human phosphor-Erk1 (pT202/pY204)/Erk2 (pT185/pY187)	Epitomics, Inc.	MAb	1∶500 (WB)	O/N	4°C	WB
Mouse anti-human β-tubulin	Sigma	MAb	1∶10000 (WB)	O/N	4°C	WB
Rat anti-mouse CD31(PECAM1) antibody	BD Pharmingen	MAb	1∶ 100 (IHC)	O/N	4°C	IHC
Rabbit anti-human Ki 67 antibody	Epitomics, Inc.	PAb	1∶1000 (IHC)	O/N	4°C	IHC
Rabbit anti-human caspase-3 (active) antibody	Epitomics, Inc.	PAb	1∶100 (IHC)	O/N	4°C	IHC
Mouse anti-human IgG1-negative control	Dako	IgG1	1∶1000 (IF)	O/N	4°C	IF
Rabbit anti-human IgG-negative control	Dako	IgG	1∶1000 (IF)	O/N	4°C	IF
Goat anti-mouse Alexa Fluor® 488 Dye Conjugate	Invitrogen	IgG	1∶1000 (IF)	45	RT	IF
Goat anti-rabbit Alexa Fluor® 488 Dye Conjugate	Invitrogen	IgG	1∶1000 (IF)	45	RT	IF
Swine anti-goat, mouse, rabbit immunoglobulins, biotinlyated, Multi-Link	Dako Pty. Ltd.	PAb	1∶150 (IHC)	45	RT	IHC
Rabbit anti-rat immunoglobulins, biotinlyated	Dako Pty. Ltd.	PAb	1∶400 (IHC)	45	RT	IHC
Goat anti-rabbit IgG-HRP	Santa Cruz Biotechnology	PAb	1∶5000 (WB)	45	RT	WB
Goat anti-mouse IgG-HRP	Santa Cruz Biotechnology	PAb	1∶5000 (WB)	45	RT	WB

**Notes:** MAb: monoclonal antibody; O/N: overnight; PAb: polyclonal antibody; RT: room temperature.

### Cell line and cell culture

The androgen-non-responsive PC-3M-luc-C6 (PC-3M-luc) CaP cell line was obtained from Xenogen Corp, USA. All tissue culture reagents were supplied by Invitrogen Australia Pty Ltd (Melbourne, VIC, Australia), unless otherwise stated. PC-3M-luc cells were cultured in RPMI-1640 supplemented with 10% (vol/vol) heat-inactivated fetal bovine serum (FBS), 50 U/mL of penicillin, and 50 U/mL of streptomycin. PC-3M-luc-scr (scrambled shRNA control), PC-3M-luc-CD44-knockdown (KD), PC-3M-luc-CD147-KD cells were grown in the same medium supplemented with additional 1 µg/mL puromycin for screening. All cell lines were maintained in a humidified incubator at 37°C and 5% CO_2_.

### Short hairpin RNA (shRNA) transfection for CD44/CD147

PC-3M-luc cells with shRNA-mediated KD of CD44 (s and v)/CD147 or a scrambled sequence control for off-target effects (PC-3M-luc-scr) were generated using a previously published method with modification [22]. Five MISSION® lentiviral transduction particles encoding for shRNAs against CD44 or CD147 and MISSION® non-target shRNA control transduction particles were used (Sigma-Aldrich, Pty Ltd, Castle Hills, NSW, Australia) (**Table S1**). Briefly, 2×10^4^ PC-3M-luc cells were cultured in 24 well plates and transduced with all five clones of lentiviral particles or the same amount of non-target shRNA control transduction particles following the manufacturer's protocol (multiplicity of infection = 2, viral transducing units/cell). The transduced clones were selected in puromycin-containing cell culture medium (0.5 µg/mL) (Invitrogen Australia Pty Ltd, Melbourne, VIC, Australia), propagated and finally transferred to the 25 cm^2^ cell culture flasks and maintained in medium containing 1.0 µg/mL puromycin for the following experiments.

### Immunofluorescence confocal microscopy analysis of PC-3M-luc and PC-3M-luc-KD cell lines

Immunofluorescence staining was performed as previously described [23]. Briefly, cells grown on glass coverslips were fixed, rinsed and incubated with various primary antibodies overnight (o/n) at 4°C: mouse anti-human CD44 monoclonal antibody (MAb) (1∶200 dilution), CD147 polyclonal antibody (PAb) (1∶100 dilution), MRP2 MAb (1∶50 dilution), rabbit anti-human MCT1 PAb (1∶200 dilution) or MCT4 PAb (1∶400 dilution). After rinsing in TBS, cells were incubated for 45 minutes in Alexa Fluor-488 goat anti-mouse or Alexa Fluor-488 goat anti-rabbit IgG (1∶1000 dilutions) at room temperature (RT). Propidium iodide (PI) (0.2 mg/L) was used to stain the nuclei. Negative controls were treated identically but incubated with either mouse or rabbit isotype control. Immunofluorescence was visualized using an FV300/FV500 Olympus laser scanning confocal microscope (Olympus, Tokyo, Japan).

### Western blotting analysis

Protein expression levels were determined by Western blotting analysis as described [24]. Briefly, whole cell lysates were separated by NuPAGE Novex 4–12% Bis-Tris gel electrophoresis and then transferred to polyvinylidene difluoride membrane. After blocking of non-specific sites with 5% skim milk, the membrane was incubated with specific antibodies at appropriate concentrations (**Table** **1**), followed by incubation with horseradish peroxidase (HRP)-conjugated secondary antibodies (goat anti-mouse or goat anti-rabbit appropriate for the host species of primary antibody) (1∶5000 dilution). Immunoreactive bands were detected using enhanced chemiluminescence (ECL) substrate (Pierce Chemical Co, Rockford, USA), and imaged using the ImageQuant LAS4000 system (GE Health care, USA). To confirm equal loading of protein lysates, membranes were stripped (Restore Western Blot Stripping Buffer, Pierce) and re-probed using mouse anti-*β*-tubulin MAb (1∶10000 dilution), then processed as above. Images were processed in Adobe Photoshop.

### Co-immunoprecipitation (IP) assay

Cell lysate containing 200 μg whole cell lysates from PC-3M-luc cells, was incubated with 2 μg of anti-CD44 or anti-CD147 antibodies (Abs) or normal serum (Ig control) o/n at 4°C. Immune complexes were isolated by precipitation using protein A/G PLUS-agarose (Santa Cruz Biotechnology, Inc, USA) for 8 h at 4°C. Beads were washed 4 times at 1000 *g* and were suspended in 20 *μ*L of NuPAGE LDS sample buffer (Invitrogen Australia Pty Ltd, Australia), then heated at 100°C for 5 minutes. Extracts were then immunoblotted with CD44 and CD147 primary antibodies, followed by incubation with HRP-conjugated secondary antibodies and detected using ECL substrate. Image analysis was as described above (Western blotting).

### MTT assay for DTX response

MTT assays were performed as described previously [24]. Briefly, cells were seeded in 96-well plates and treated with a range of concentrations (0.001–1000 nM) of DTX diluted in 100% ethanol, and a vehicle control. After 72 h incubation, the medium was replaced with fresh medium containing 0.5 mg/mL MTT [3-(4,5-dimethylthiazol-2-yl)-2,5-diphenyltetrazolium bromide]. After 4 h, the supernatants were removed and the resulting MTT formazan solubilized in DMSO and measured spectrophotometrically at 562 nm on a BIO-TEK microplate reader (Bio-Rad, Hercules, CA, USA). Results represent the OD ratio of DTX-treated and vehicle-treated cells. The growth inhibition curve was generated using the GraphPad Prism 4 Program (GraphPad, San Diego, CA, USA). Absolute IC_50_ values were calculated using the intersection of the 50% normalised drug response and the growth inhibition curves for each cell line, to find the x axis values for IC_50_ DTX concentration (nM).

### Colony forming assays

PC-3M-luc and PC-3M-luc-KD cells were used for colony forming assays as described previously with minor modifications [25]. Briefly, 1500 cells/dish were seeded in 10 cm dishes for 48 h at 37°C, 5% CO_2_ and then treated with a fixed dose of DTX at 3.5 nM final concentration (1/2 dose of the lowest IC_50_ from the MTT assay in four CaP cell lines) or the same volume of vehicle control (100% ethanol). After 3 days treatment, the DTX-containing media was replaced with fresh media and all cultures were incubated for an additional 7 days until colonies were large enough to be clearly discerned. The colonies, defined as groups of >50 cells, were scored manually with the aid of an Olympus INT-2 inverted microscope (Tokyo, Japan). The average number of colonies were plotted (Mean ± SD, n = 3).

### Matrigel invasion assay

Invasive ability of CaP cell lines was determined using commercial matrigel and control transwell chambers (BD Bioscience, NSW, Australia). Briefly, 2×10^4^ PC-3M-luc, PC-3M-luc-scr, PC-3M-luc-CD44-KD and PC-3M-luc-CD147-KD CaP cells in 500 µL serum-free medium were added to each transwell insert and 750 µL complete medium was added to the outer well to provide chemoattractant and prevent dehydration. Cells were incubated at 37°C in 5% CO_2_ for 24 h and then stained with a Diff-Quik staining kit (Allegiance Healthcare Corp, McGraw Park, Illinois, USA). Excess dye was washed away with tap water and the number of stained cells that invaded through matrigel or control inserts was counted in five high power fields (hpf) by light microscopy (Leica microscope, Nussloch, Germany). Invasive potential was calculated as follows: % Invasion  =  [(Mean cells invading through matrigel insert membrane)/(Mean cells migrating through control insert membrane)] ×100%. Cell invasion rates were plotted, with mean and SD (n = 3).

### Subcutaneous (s.c.) xenograft animal model

Male, 6–8 weeks old Balb/c nude mice (Animal Resources Centre, Western Australia) were housed under specific pathogen-free conditions in facilities approved by the University of New South Wales (UNSW) Animal Care and Ethics Committee (ACEC) and manipulations were performed in laminar flow cabinets. The ACEC specifically approved this study (approval ID is 10/121A). Mice were kept at least 1 week before experimental manipulation. All mice remained healthy and active during the experiment. As described previously [26], cultured PC-3M-luc, PC-3M-luc-scr, PC-3M-luc-CD44-KD or PC-3M-luc-CD147-KD CaP cells (1.5 x 10^6^/injection) in 100 µL DPBS were implanted subcutaneously in the right rear flank region of mice (n = 10 mice/per group). Tumor progression was documented weekly by measurements using callipers, and tumor volumes were calculated as follows: length × width × height ×0.52 (in millimetres) [27] for up to 8 weeks. Upon sacrifice, primary tumors and local regional lymph nodes were removed for histological examination.

### DTX response in CaP s.c. animal model

After establishing s.c. models with CaP cell lines, when average tumor size reached 30±10 cm^3^ in each subgroup (n = 10/subgroup), 5 mice (n = 5) were treated with 25 mg/kg DTX continuously for 3 weeks by i.p. injection, and 5 mice (n = 5) were treated with vehicle control (saline). Tumor growth was calculated by measurements using calipers as published [27].

### Mouse tissues and histology

All tissues were either formalin fixed or snap frozen. Hematoxylin and eosin (H&E)-stained sections were reviewed to assess tumor structure. Tumor xenografts and local regional lymph nodes were collected at the end of experiments and processed for histology, immunohistochemistry and TUNEL assay. Five-micron frozen sections of fresh tumor samples were used for CD31 and CD44 immunostaining.

### Immunohistochemistry

Standard immunoperoxidase procedures were used to visualize CD147, Ki-67, and caspase-3 (active) as previously published [28]. Briefly, paraffin sections were dewaxed and rehydrated, then incubated with primary antibodies (Abs) anti-CD147 (1∶200), Ki-67 (1∶1000 dilution) and caspase-3 (active) (1∶100 dilution), respectively o/n at 4°C. Slides were then incubated with swine anti-goat, mouse, rabbit biotinylated IgG second antibody (1∶150 dilution) for 45 minutes at RT and with streptavidin/HRP solution (1∶300 dilution) for 30 minutes at RT. Sections were finally developed with 3,3′ diaminobenzidine (DAB) substrate solution (Sigma-Aldrich, Pty Ltd, Castle Hills, NSW, Australia), then counterstained with hematoxylin; positive cells appeared brown. Control slides were treated in an identical manner, and using isotype Abs or omitting primary antibody as a negative control.

CD44 and CD31 immunostaining on frozen sections were performed as previously published (28). Sections were incubated with mouse anti-human CD44 (1∶200 dilution) or rat anti-mouse CD31 MAb (1∶100 dilution) o/n at 4°C. Sections were incubated with rabbit anti-mouse/rat biotinylated IgG (1∶200 dilution) for 45 minutes at RT, and then with conjugated streptavidin/HRP (1∶200 dilution) for another 30 minutes. Sections were developed by using DAB solution (Sigma-Aldrich, Pty Ltd, Castle Hills, NSW, Australia), and counterstained with hematoxylin. For negative controls, sections were stained with the isotype MAb or with omitting primary antibodies.

### TUNEL assay for apoptotic cells *in vivo*

Apoptosis was assessed on tumor xenograft tissues using the TUNEL method with the TdT-fragEL in situ apoptotic detection kit (Calbiochem, San Diego, CA, USA) according to the manufacturer's instructions. The specificity of TUNEL reactivity was confirmed with appropriate negative (TdT omitted from the labeling mix) and positive (treated HL-60 slides provided by the company) controls. Slides were examined using a Leica light microscope (Nussloch, Germany).

### Assessment of immunostaining

Staining intensity (0–3) was assessed using light microscopy (Leica, Germany) and a x40 objective. The criteria used for assessment were as previously reported [29], where: 0 (negative, <25%); 1+ (weak, 25–50%); 2+ (moderate, 50–70%); 3+ (strong, >75%) of the tumor cells stained. Evaluation of tissue staining was done, independently, by three experienced observers (JH, JB and YL). All specimens were scored blind and an average of grades was taken.

### Statistical analysis

All numerical data were expressed as the average of the values obtained, and the standard deviation (SD) was calculated. Data from different groups were compared using the two-tail student's t test. All *P* values were 2-sided. The statistical analysis of immunostaining intensity in animal xenografts was performed as described in a recent publication [28]. One-way ANOVA, followed by the Dunnett's post hoc test was performed to determine the significance of differences between the growth curves in s.c. model for tumor volume changes. *P*<0.05 was considered significant. All numerical statistical analyses were performed using the GraphPad Prism 4.00 package (GraphPad, San Diego CA).

## Results

### Expression of CD44, CD147, MCT4 and MRP2 in CD44 or CD147-KD and control cell lines

**Figure 1:**
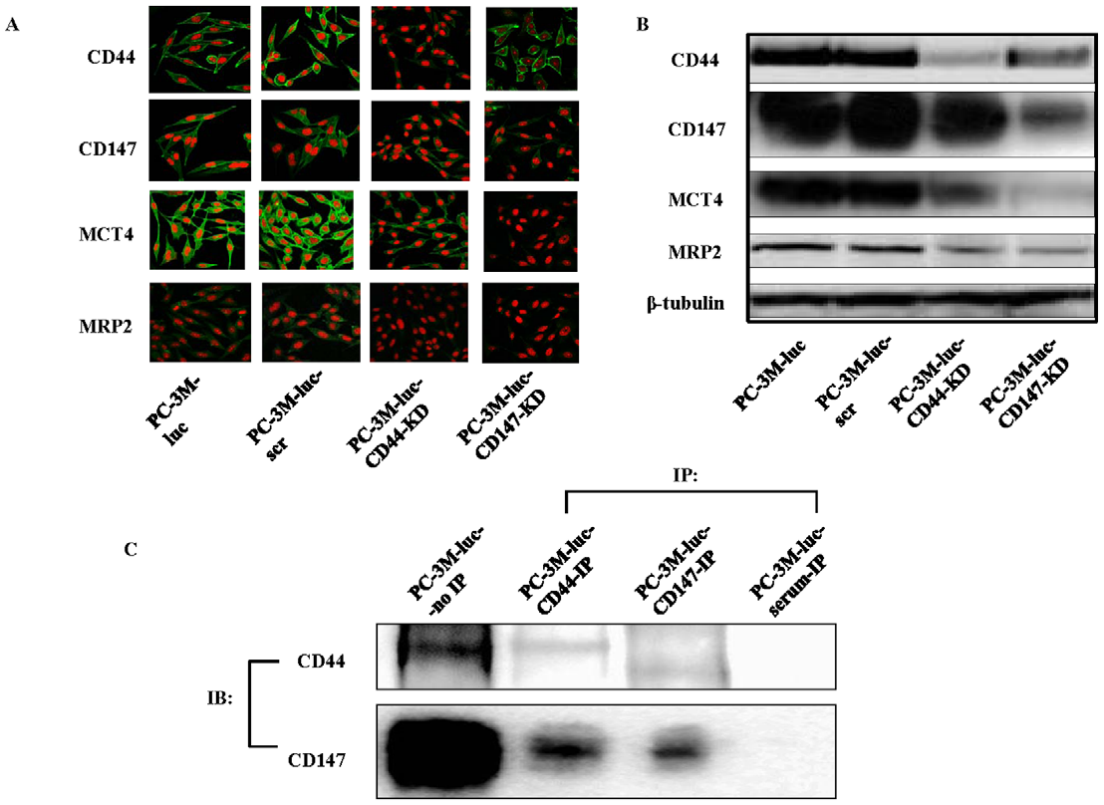
The expression of CD44 and CD147 in PC-3M-luc cells and the effects of reducing CD44 or CD147 on each other and/or MRP2 and MCT4 in CaP cell lines. Representative confocal images of CD44, CD147, MCT4 and MRP2 (green) immunofluorescence after knocking down CD44 or CD147 (A). Nuclei are stained with PI (red). Magnification: all images ×400. Representative western blot is shown to confirm the immunofluorescence staining (B). β-tubulin was used as a loading control. scr: scrambled shRNA control. PC-3M-luc cell lysates immunoprecipitated with anti-CD44, anti-CD147 antibody, and normal serum (Ig) followed by immunoblotting with either CD44 or CD147 antibodies (C). IB: immunoblotting; IP: Immunoprecipitation.

PC-3M-luc and PC-3M-luc-scr CaP cell lines showed strong positive staining for CD44, CD147, MCT4 and MRP2 (**Fig.** **1A**). Following CD44 knock down, the reduction in CD44 expression was also associated with a concomitant reduction in the levels of expression of CD147, MCT4 and MRP2 (**Fig.** **1A**). Similarly, after knocking down CD147, the levels of CD147 expression were reduced and a concomitant reduction in the levels of expression of CD44, MCT4 and MRP2 was also seen (**Fig.** **1A**). No detectable staining was seen in the cells incubated with isotype controls (data not shown). The immunofluorescence staining results in different CaP cell lines are summarized in **Table** **2**. The immunofluorescence results for the expression of CD44, CD147, MCT4 and MRP2 in CaP cell lines were further confirmed by western blotting (**Fig.** **1B**). PC-3M-luc cell lysates were also immunoprecipitated with either anti-CD44 antibody, anti-CD147 antibody or normal serum (Ig), and immunoblotted with CD44 and CD147 Abs (**Fig.** **1C**). Both CD44 (90 KDa) and CD147 (∼50 KDa) bands were detected in anti-CD44 and anti-CD147 antibody IP lysates respectively, but not in the Ig precipitated lysate.

**Table 2:** Immunostaining for CD44, CD147, MCT4 and MRP2 in KD and control prostate cancer (CaP) cell lines.

Cell Line	CD44	CD147	MCT4	MRP2
PC-3M-luc	2–3	2–3	3	1–2
PC-3M-luc-scr	2–3	2–3	3	1–2
PC-3M-luc-CD44-KD	0–1	1–2	1–2	0–1
PC-3M-luc-CD147-KD	1–2	0–1	0	1

**Notes:** **Immunofluorescence staining:** 0 =  negative; 1 =  weak; 2 =  moderate; 3 =  strong.

KD: knock down; scr: scrambled shRNA control.

### Knock down of CD44 or CD147 sensitizes monolayer CaP cells to DTX treatment *in vitro*

**Figure 2:**
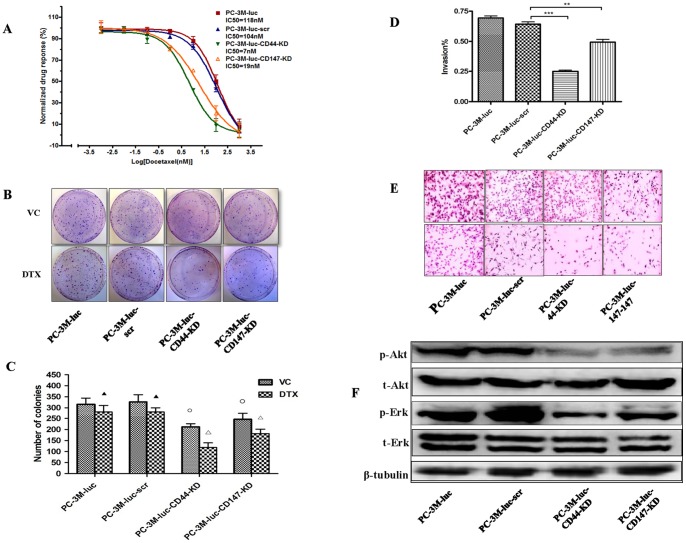
MTT, colony assays, matrigel invasion and PI3K/Akt or MAPK/Erk related signaling proteins after CD44 or CD147 knock down in PC-3M cells. PC-3M-luc, PC-3M-luc-scr, PC-3M-luc-CD44-KD and PC-3M-luc-CD147-KD CaP cells treated with DTX (0.001–1000 nM) showed variable response. Sensitivity of CD44/CD147-KD cells to different concentrations of DTX compared to controls was obviously increased by MTT assay (**A**) (*P*<0.01). Four CaP cell lines were seeded in 10 cm dishes and treated with a fixed DTX dose (3.5 nM) for 3 d. Following treatment, cells were cultured in growth medium for 7 d. Results are presented as the number of colonies formed. Typical images are shown for colony growth in CaP cell lines treated with VC or DTX (**B**). Typical results of DTX sensitivity in colonies of CaP cell lines are shown (**C**): “▴” indicates no significant difference in the average number of colonies between the DTX treated cells and VC treated cells in PC-3M-luc/PC-3M-luc-scr cell lines (*P*≥0.05); “○” indicates significant difference in the average number of colonies between the DTX treated cells and VC treated cells in PC-3M-luc-CD44-KD/PC-3M-luc-CD147-KD cell lines (*P*<0.05); “Δ” indicates significant difference in the average number of colonies between DTX treated cells in PC-3M-luc-CD44-KD/PC-3M-luc-CD147-KD cell lines and VC treated cells in PC-3M-luc/PC-3M-luc-scr cell lines (*P*<0.05). Matrigel invasion assay was used to study the change in invasion potential in PC-3M-luc, PC-3M-luc-scr and PC-3M-luc-CD44/CD147-KD cells. Invasive potential was significantly reduced to 25% and 50% in PC-3M-luc-CD44-KD and PC-3M-luc-CD147-KD respectively, compared to 69% and 64% in PC-3M-luc and PC-3M-luc-scr, respectively (*P*<0.01) (**D**). Representative light microscopy images for CaP cell invasion (**E**). Four signal transduction molecules (p-Akt, t-Akt, p-Erk and t-Erk) were assessed to investigate the relationship between CD44, CD147 and cell signalling pathways. The levels of p-Akt and p-Erk were reduced in KD cell lines compared to wild-type and scr controls. Representative results are shown (**F**). All results were from three independent experiments (Mean ± SD, n = 3). DTX: docetaxel; KD: knock down; p-Akt: phosphorylated-Akt; p-Erk: phosphorylated-Erk; scr: scrambled shRNA control; t-Akt: total-Akt; t-Erk: total-Erk; VC: vehicle control; * indicates 0.01<*P*<0.05; ** indicates 0.001<*P*<0.01; *** indicates *P*<0.001.

KD (PC-3M-luc-KD-CD44 and PC-3M-luc-KD-CD147) and control (PC-3M-luc and PC-3M-luc-scr) CaP cell lines with different levels of CD44 and CD147 expression responded differently to DTX treatment. The IC_50_ values (the dose for obtaining 50% cell killing) strongly correlated with the levels of CD44 and CD147 expression (**Fig.** **2A**). Accordingly, PC-3M-luc and PC-3M-luc-scr control cells (high levels of CD44 and CD147) were the least sensitive (IC_50_: 118 and 104 nM, respectively), while PC-3M-luc-CD44-KD and PC-3M-luc-CD147-KD cells (low levels of CD44 and CD147) were very sensitive to DTX treatment (IC_50_: 7 and 19 nM, respectively). Significant differences (*P*<0.05) in the IC_50_ was observed between PC-3M-luc-CD44-KD/PC-3M-luc-CD147-KD cells and PC-3M-luc/PC-3M-luc-scr cells.

### Knock down of CD44 or CD147 reduces clonogenic ability and sensitizes CaP colonies to DTX treatment

To investigate whether KD of CD44 and CD147 affect the clonogenicity of either alone or combined with DTX treatment, we assessed KD and control CaP cells in culture. The number of colonies was significantly decreased either with vehicle control or with DTX treatment in PC-3M-luc-CD44 or CD147-KD cells compared with PC-3M-luc or PC-3M-luc-scr cells, while there was no significant difference (*P*≥0.05) between the number of colonies generated from PC-3M-luc and PC-3M-luc-scr cells. Significant differences (*P*<0.05) in the average number of colonies was observed 1) between DTX treated cells and VC treated cells in PC-3M-luc-CD44-KD/CD147-KD cell lines; 2) between DTX treated PC-3M-luc-CD44-KD/CD147-KD cells and VC treated PC-3M-luc/PC-3M-luc-scr cells. Representative images are shown in **Fig.** **2B**. The DTX response in PC-3M-luc-CD44 or CD147-KD cell lines was greater than for PC-3M-luc and PC-3M-luc-scr cell lines (**Fig.** **2C**). The clonogenicity (average DTX-treated colonies/average vehicle control-treated colonies %) was 89%, 84%, 56% and 74% for PC-3M-luc, PC-3M-luc-scr, PC-3M-luc-CD44-KD, and PC-3M-luc-CD147-KD cell lines, respectively.

### Knock down of CD44 or CD147 reduces CaP cell invasion

After knocking down CD44 or CD147, cell invasion was significantly reduced for both PC-3M-luc-CD44-KD (*P*<0.001) and PC-3M-luc-CD147-KD cells (*P*<0.01) compared with PC-3M-luc and PC-3M-luc-scr control cells (**Fig.** **2D**). A relatively greater reduction in invasion ability was found in PC-3M-luc-CD44-KD cells than in PC-3M-luc-CD147-KD cells (**Fig.** **2D**). The percentage invasion for PC-3M-luc, PC-3M-luc-scr, PC-3M-luc-CD44-KD and PC-3M-luc-CD147-KD cells was 70%, 62%, 22%, and 47% respectively (**Fig.** **2D**). Representative images for each cell line are shown in **Fig.** **2E**.

### PI3K/Akt and MAPK/Erk signaling pathways are related to the expression of CD44 and CD147 in CaP cells

After knocking down CD44 or CD147, we found that the expression of p-Akt and p-Erk were both downregulated in PC-3M-luc-CD44 or CD147-KD cells compared to PC-3M-luc and PC-3M-luc-scr control cells, with more significant reduction in PC-3M-luc-CD44-KD cells; there was no obvious change in t-Akt and t-Erk expression in all CaP cell lines (**Fig.** **2F**).

### Knock down of CD44 or CD147 affects tumorigenicity, lymph node metastases and DTX sensitivity in a s.c. xenograft model

**Figure 3:**
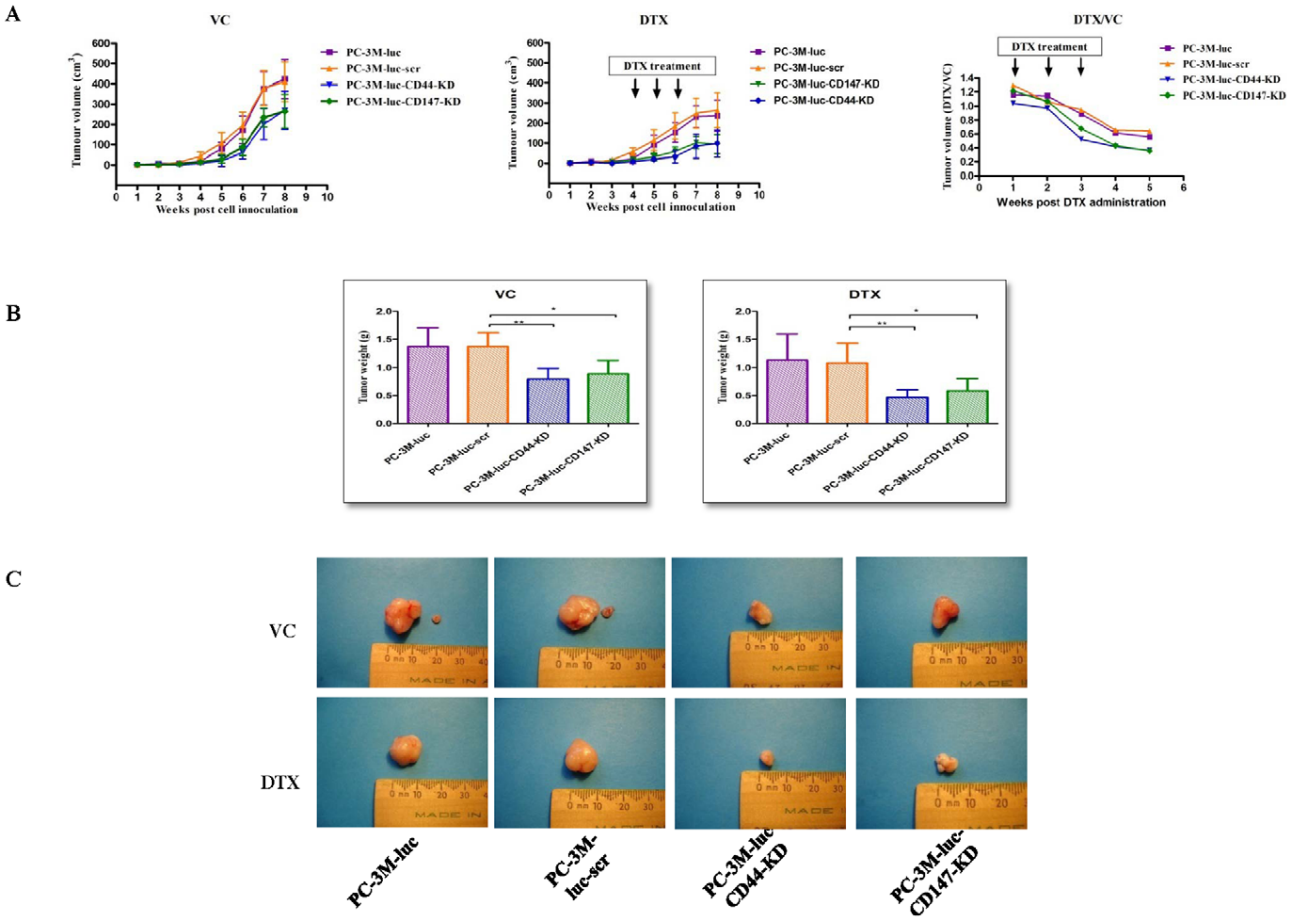
*In vivo* effects of CD44- and CD147-KD on tumorigenicity and sensitivity to DTX in s.c xenograft models. Tumor growth curves for PC-3M-luc, PC-3M-luc-scr, PC-3M-luc -CD44-KD and PC-3M-luc-CD147-KD xenografts are shown either with VC (the plot on the left) or with DTX treatments (the plot in the middle). The ratio of DTX treated versus VC treated tumor volumes (DTX/VC) was plotted from the start of treatment to the end of experiment (shown in the plot on the right) (**A**). At the end of experiments, tumor weight from PC-3M-CD44-KD and PC-3M-luc-CD147-KD group mice was obviously reduced compared to that from PC-3M-luc and PC-3M-luc-scr group mice with VC and DTX treatments (*P*<0.05) (**B**). Representative images for tumor sizes and lymph node metastases from different groups and treatments are shown (**C**). DTX: docetaxel; KD: knock down; scr: scrambled shRNA control; VC: vehicle control. * indicates 0.01< *P*<0.05; ** indicates 0.001< *P*<0.01; *** indicates *P*<0.001.

As shown in the tumor growth graphs in **Fig.** **3A**, compared with scr control, the weekly measurements of CD44 and CD147 KD xenografts, treated either with vehicle control (VC) or DTX (25 mg/kg), showed a significantly reduced tumor growth rate in both VC (*P*<0.05) and DTX-treated groups (*P*<0.05) (on the left and middle panels). There were no significant differences in the tumor growth rate of PC-3M-luc wild-type and PC-3M-luc-scr xenografts in either VC or DTX treated groups (*P*>0.05). In VC-treated xenografts, a slightly stronger regression of tumor growth was seen in CD44 KD xenografts compared with CD147 KD xenografts, while no obvious difference was found between growth of CD44 KD and CD147 KD tumors (*P*>0.05). In the DTX-treated xenografts, the tumor xenografts from the four CaP cell lines had smaller tumor volumes compared to corresponding VC-treated xenografts at all time points (*P*<0.05). Slightly more tumor regression is seen in DTX-treated CD44-KD xenografts but no significant difference is found between CD44-KD and CD147-KD growth curves (*P*>0.05). In addition, we compared the ratio of tumor volumes between DTX and VC treated xenografts (DTX/VC) (Fig. 3A, right panel). The ratio of CD44-KD and CD147-KD tumor volumes (DTX/VC) decreased faster in response to DTX or VC treatment, suggesting that the KD of either CD44 or CD147 can induce suppression of tumor development, compared to the scr and wild-type xenografts.

We also evaluated tumor weight changes in each group at completion of the experiments (8 weeks post cell inoculation) (**Fig.** **3B** **)**. We found no significant difference in weight between the parental and scramble-transfected cell lines, showing that the cells' *in vivo* charactersitics was not altered by the transfection process (**Fig.** **3B and C**). At this time point, CD44-KD and CD147-KD xenografts both showed significantly reduced tumor weights compared to control groups (789±192 mg for PC-3M-luc-CD44-KD and 892±238 mg for PC-3M-luc-CD147-KD versus 1369±117 mg for PC-3M-luc and 1376±250 mg for PC-3M-luc-scr, respectively). This represented 58% and 65% weight reductions in KD-treated versus scr-control groups (*P*<0.05), respectively (**Fig.** **3B and C**). In addition, we observed an obvious decrease in overall body weight in the DTX-treated mice compared to the untreated control mice (*P*<0.05, unpublished data). There was also an obvious delay in weight gain after DTX treatment in all mice (unpublished data).

All mice from control groups without DTX treatment developed regional lymph node metastases; no lymph node metastases were seen in CD44- or CD147-KD or control mice treated with DTX (**Fig. S1**). Tumor volumes of mice with KD xenografts were less than control groups, and tumor volumes of DTX-treated mice were less than VC-treated mice. Typical tumor sizes including lymph node metastases are shown in **Fig.** **3C**.

### Tumor xenografts histology following CD44 or CD147-KD with or without DTX treatment

**Figure 4:**
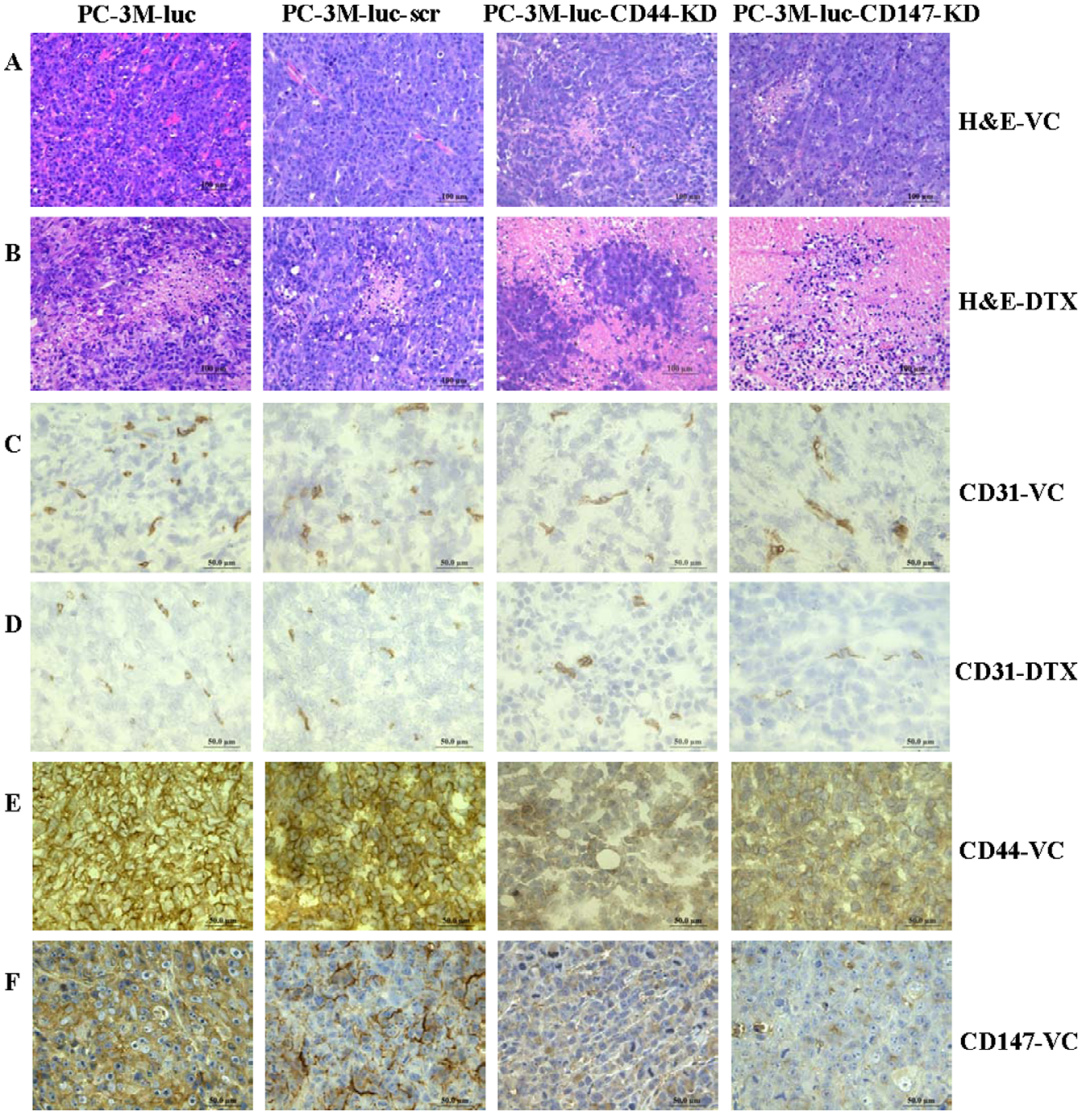
Histology and CD31, CD44 and CD147 expression of s.c. xenografts. Morphological changes are shown in PC-3M-luc-CD44-KD and PC-3M-luc-CD147-KD xenografts with VC treatment (**A**). Obvious targeted lesions are shown in PC-3M-luc-CD44-KD and PC-3M-luc-CD147-KD xenografts with DTX treatment compared to the xenografts from PC-3M-luc and PC-3M-luc-scr cell lines (**B**). Low to medium CD31 expression is shown in PC-3M-luc-CD44-KD and PC-3M-luc-CD147-KD treated with VC and high CD31 expression is shown in PC-3M-luc and PC-3M-luc-src xenografts treated with VC (*P*<0.05) (**C**). Markedly reduced CD31 expression is shown in PC-3M-luc-CD44-KD and PC-3M-luc-CD147-KD xenografts treated with DTX and medium CD31 expression is shown in PC-3M-luc and PC-3M-luc-scr xenografts treated with DTX (*P*<0.05) (**D**). High CD44 expression is shown in PC-3M-luc and PC-3M-luc-scr xenografts; medium CD44 expression is seen in PC-3M-luc-CD147-KD xenografts; low CD44 expression is seen in PC-3M-luc-CD44-KD xenografts (*P*<0.05) (**E**). High CD147 expression is shown in PC-3M-luc and PC-3M-luc-scr xenografts; low CD147 expression is seen in PC-3M-luc-CD44-KD xenografts; very low CD147 expression is seen in PC-3M-luc-CD147-KD xenografts (*P*<0.05) (**F**). Brown color staining indicates positive while blue hematoxylin stains nuclei. Magnification ×400 in all images. DTX: docetaxel; KD: knock down; scr: scrambled shRNA control; VC: vehicle control.

To compare the histology of each group, tumors from control mice (PC-3M-luc- and PC-3M-luc-scr-xenografts) and KD mice (PC-3M-luc-CD44-KD- and PC-3M-luc-CD147-KD-xenografts) treated with VC and DTX were harvested at the end of the experiments, paraffin embedded, sectioned and stained with H&E. With light microscopy (**Fig.** **4**), PC-3M-luc and PC-3M-luc-scr tumor xenografts in VC treated groups consisted of tightly packed cells. Loosely packed or dispersed tumor cells associated with areas of cell death were apparent in both PC-3M-luc-CD44-KD and PC-3M-luc-CD147-KD xenografts (**Fig.** **4A**). In DTX-treated groups, the tumors were less densely packed with necrotic and apoptotic regions in all groups. This was greater in PC-3M-luc-CD44-KD and PC-3M-luc-CD147-KD xenografts, compared to PC3-3M-luc or PC-3M-luc-scr tumors (**Fig.** **4B**).

### Assessment of tumor microvascular density in CD44 or CD147-KD tumor xenografts with/without DTX treatment

To assess the effect of CD44 or CD147-KD on vascular density in different xenografts, frozen sections of tumors were stained with CD31 MAb. Representative images for CD31 expression in VC-treated mice (**Fig.** **4C**) and DTX-treated mice (**Fig.** **4D**) are shown. Microvascular density (MVD) (number of CD31 positive cells/ hpf) was high in PC-3M-luc and PC-3M-luc-scr xenografts treated with VC; this was decreased in VC treated PC-3M-luc-CD44-KD and PC-3M-luc-CD147-KD xenografts; with the greatest reduction in DTX treated in PC-3M-luc-CD44-KD and PC-3M-luc-CD147-KD xenografts (**Fig.** **4C and D**).

The numbers of CD31^+^ vessels/per area in PC-3M-luc, PC-3M-luc-scr, PC-3M-luc-CD44-KD and PC-3M-luc-CD147-KD groups were 16–20/ hpf, 9–13/ hpf, 6–10/ hpf, 5–8/ hpf, respectively in VC-treated mice. The numbers of CD31^+^ vessels/per area in PC-3M-luc, PC-3M-luc-scr, PC-3M-luc-CD44-KD and PC-3M-luc-CD147-KD groups were 14–19/ hpf, 9–12/ hpf, 6–8/ hpf, 2–5/ hpf, respectively in DTX-treated mice. The CD31 immunostaining results are summarized in **Table** **3**. The difference in staining intensity for CD31 between PC-3M-luc-CD44-KD/PC-3M-luc-CD147-KD and PC-3M-luc/PC-3M-luc-scr cell lines was significant (*P*<0.05). PC-3M-luc-CD147-KD xenografts had least CD31 expression in DTX-treated mice.

**Table 3:** The intensity of immunohistochemical staining of CD44, CD147, CD31, Ki-67, Caspase-3(active), TUNEL in tumor xenografts from PC-3M-luc, PC-3M-luc-scr, PC-3M-luc-CD44-KD and PC-3M-luc-CD147-KD with DTX and/or veihicle treatment.

	PC-3M-luc		PC-3M-luc-scr		PC-3M-luc-CD44-KD		PC-3M-luc-CD147-KD	
	VC	DTX	VC	DTX	VC	DTX	VC	DTX
**CD44**	95–100%	N/A	95–100%	N/A	30–40%	N/A	40–50%	N/A
	+++		+++		+		++	
**CD147**	80–90%		70–80%		20–30%		∼20%	
	+++		+++		+		+	
**CD31**	16–20/hpf	14–19/hpf	9–13/hpf	9–12/hpf	6–10/hpf	6–8/hpf	5–8/hpf	2–5/hpf
	+++	+++	+++	+++	++	+	++	+
**Ki-67**	70–80%	60–70%	80–90%	60–70%	60–80%	50–60%	60–70%	50%
	+++	+++	+++	+++	++	+	++	+
**Caspase-3 (a)**	0–5/hpf	8–12/hpf	1–7/hpf	8–13/hpf	7–10/hpf	30–35/hpf	12–17/hpf	20–25/hpf
	−	+	−	+	+	++	+	++
**TUNEL**	0–5/hpf	11–15/hpf	0–6/hpf	15–19/hpf	17–21/hpf	28–35/hpf	12–18/hpf	30–36/hpf
	−	++	−	++	+	+++	+	++

**Notes:** a: active; DTX: docetaxel; hpf: high power fields; KD: knocking down; N/A: not available; scr: scrambled shRNA control; VC: vehicle control.

### CD44 and CD147 expression in tumor xenografts after knock down of CD44 or CD147

At the end of experiments, expression of CD44 and CD147 was examined for the VC-treated xenografts from PC-3M-luc, PC-3M-luc-scr, PC-3M-luc-CD44-KD and PC-3M-luc-CD147-KD cells with CD44 and CD147 Abs. Our results indicated high levels of CD44 expression in PC-3M-luc and PC-3M-luc-scr xenografts (95–100%, +++); intermediate levels of CD44 in CD147 KD xenografts (40–50%, ++); low levels of CD44 in CD44 KD xenografts (30%–40%, +) (**Fig.** **4E** **,** **Table** **3**). A significant difference in staining intensity for CD44 expression was found between PC-3M-luc-CD44-KD/PC-3M-luc-CD147-KD and PC-3M-luc/PC-3M-luc-scr cell lines (*P*<0.05). High levels of CD147 were observed in PC-3M-luc (80–90%, +++) and PC-3M-luc-scr xenografts (70–80%, +++); low levels of CD147 in CD44 KD xenografts (20–30%, +); very low levels of CD147 in CD147 KD xenografts (∼20%, +) (**Fig.** **4F** **,** **Table** **3**). The difference in staining intensity for CD147 expression between PC-3M-luc-CD44-KD/PC-3M-luc-CD147-KD and PC-3M-luc/PC-3M-luc-scr cell lines was significant (*P*<0.05). These results suggest that CD44 and CD147-KD can significantly reduce CD44/CD147 expression and affect each other in PC-3M-luc xenografts.

### Cell proliferation, cell death and apoptosis in tumor xenografts after CD44 or CD147-KD with or without DTX treatment

**Figure 5:**
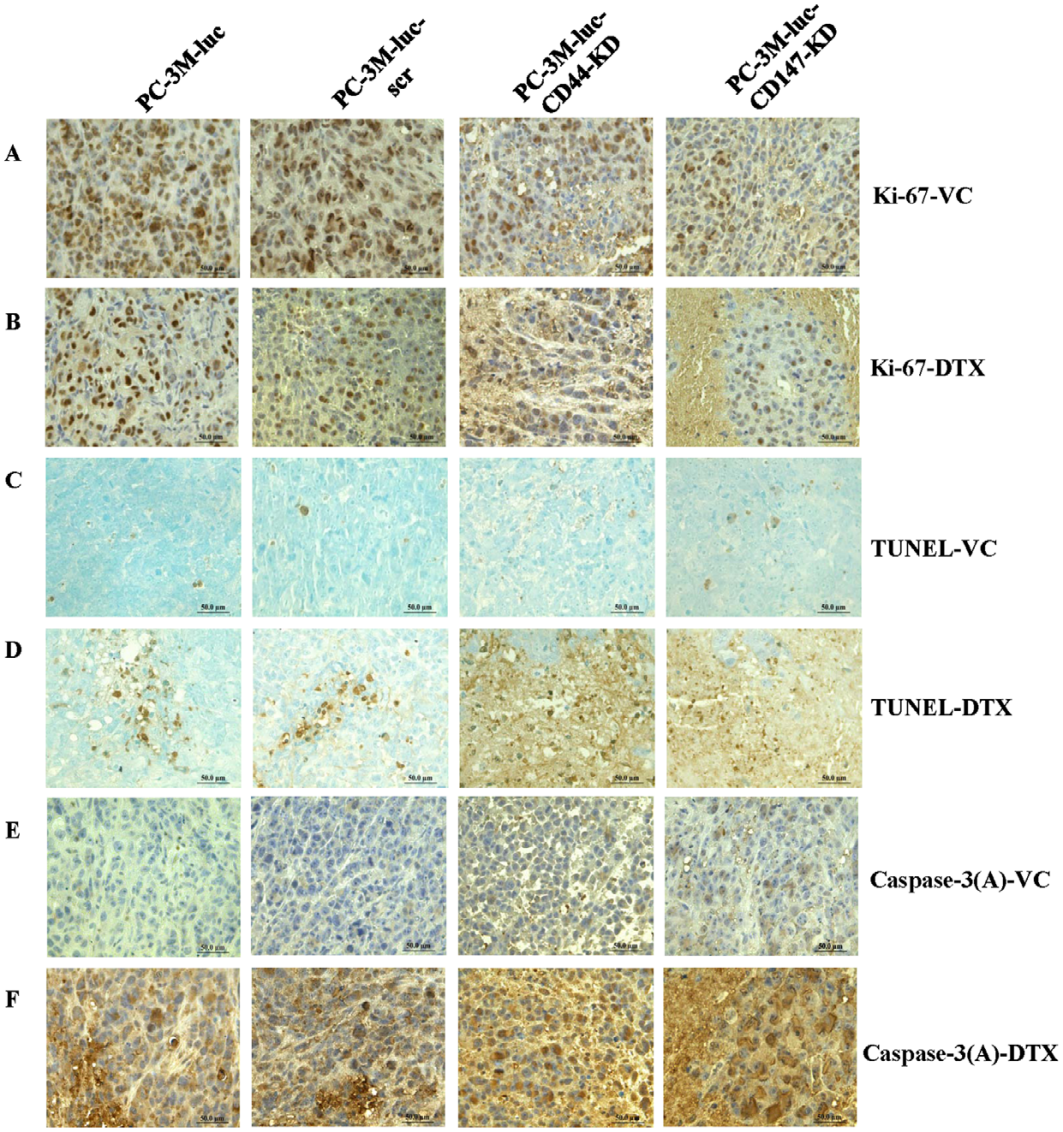
Ki-67, TUNEL, and Caspase-3 (active) expression at the end of experiments after knocking down CD44/CD147 and treatment with DTX. Very high expression of Ki-67 is seen in PC-3M-luc and PC-3M-luc-scr xenografts treated with VC; medium expression Ki-67 is seen in PC-3M-luc-CD44-KD and PC-3M-luc-CD147-KD xenografts treated with VC (*P*<0.05) (**A**). Medium to high expression Ki-67 is seen in PC-3M-luc and PC-3M-luc-scr xenografts treated with DTX; low expression Ki-67 is seen in PC-3M-luc-CD44-KD and PC-3M-luc-CD147-KD xenografts treated with DTX (*P*<0.05) (**B**). No TUNEL-positive cells are seen in PC-3M-luc and PC-3M-luc-scr xenografts treated with VC; low TUNEL-positive cells are seen in PC-3M-luc-CD44-KD and PC-3M-luc-CD147-KD xenografts treated with VC (**C**). Medium TUNEL-positive cells are seen in PC-3M-luc and PC-3M-luc-scr xenografts treated with DTX; high TUNEL-positive cells are seen in PC-3M-luc-CD44-KD and PC-3M-luc-CD147-KD xenografts treated with DTX (*P*<0.05) (**D**). Very low caspase-3 (a) cells are seen in PC-3M-luc and PC-3M-luc-scr xenografts treated with VC; low to medium caspase-3 (a) cells are seen in PC-3M-luc-CD44-KD and PC-3M-CD147-KD xenografts treated with VC (*P*<0.05) (**E**). High caspase-3 (a) cells are seen in PC-3M-luc and PC-3M-luc-scr xenografts treated with DTX; very high caspase-3 (a) cells are seen in PC-3M-luc-CD44-KD and PC-3M-luc-CD147-KD treated with DTX (*P*<0.05) (**F**). The brown color indicates nuclear staining for Ki-67 and caspase-3 (a) antibodies is brown; blue indicates nuclear staining with hematoxylin. TUNEL positive cells are brown counterstained with methyl green nuclear staining. Magnification ×400 in all images. a: active; DTX: docetaxel; KD: knock down; scr: scrambled shRNA control; VC: vehicle control.

To investigate whether knock down of CD44/CD147 in CaP cells affect proliferative potential either alone or in response to DTX *in vivo*, tumor sections from nude mice were assessed for Ki-67 expression. At the end of experiments (8 weeks), high numbers of Ki-67^+^ tumor cells were seen in PC-3M-luc (80–90%) and PC-3M-luc-scr (70–80%) xenografts; modest Ki-67^+^ tumor cells in PC-3M-luc-CD44-KD (60–80%) and PC-3M-luc-CD147-KD (60–70%) xenografts with VC-treatment suggesting that the KD has limited effects on the proliferation of the CaP cells. The difference in staining intensity for Ki-67^+^ cells between PC-3M-luc-CD44-KD/PC-3M-luc-CD147-KD and PC-3M-luc/PC-3M-luc-scr cell lines was significant (*P*<0.05). DTX treatment successfully reduced the number of proliferative cells in all groups, and but with small differences between the control and the KD groups (**Table** **3**). Representative images are shown in **Fig.** **5A and B**.

To investigate whether apoptosis may be involved in the targeted lesions of tumor xenografts, TUNEL assay was used. Representative results are shown in **Fig.** **5C and D** **.** At the end of experiments, TUNEL-positive tumor cells from PC-3M-luc-CD44-KD (28–35/ hpf) and PC-3M-luc-CD147-KD (30–36/ hpf) xenografts in DTX-treated groups displayed typical apoptotic cell morphology with nuclear chromatin condensation and fragmentation. Tumor cells from PC-3M-luc (11–15/ hpf) and PC-3M-luc-scr (15–19/ hpf) xenografts with DTX-treatment showed fewer apoptotic cells while tumors from PC-3M-luc-CD44-KD (17–21/ hpf) and PC-3M-luc-CD147-KD (12–18/ hpf) xenografts with VC-treated groups showed few apoptotic cells; almost no apoptotic cells were found in PC-3M-luc and PC-3M-luc-scr xenografts for VC-treated mice (**Fig.** **5C and D** **,** **Table** **3**). The significant difference in staining intensity for TUNEL-positive areas was seen between PC-3M-luc-CD44-KD/PC-3M-luc-CD147-KD and PC-3M-luc/PC-3M-luc-scr cell lines (*P*<0.05). We observed very high levels of caspase-3 (active)^+^ cells in PC-3M-luc-CD44-KD (30–35/ hpf) and PC-3M-luc-CD147-KD (20–25/ hpf) xenografts after DTX treatment; high level of caspase-3 (active)^+^ cells in PC-3M-luc (8–12/ hpf) and PC-3M-luc-scr (8–13/ hpf) xenografts with DTX treatment; medium level of caspase-3 (active)^+^ cells in PC-3M-luc-CD44-KD (7–10/ hpf) and PC-3M-luc-CD147-KD (12–17/ hpf) with VC treatment; only few caspase-3 (active)^+^ cells in PC-3M-luc (0–5/ hpf) and PC-3M-luc-scr (1–7/ hpf) with VC treatment (**Fig.** **5E and F** **,** **Table** **3**). The difference in staining intensity for caspase-3 (active) between PC-3M-luc-CD44-KD/PC-3M-luc-CD147-KD and PC-3M-luc/PC-3M-luc-scr cell lines was significant (*P*<0.05). Overall the regression of s.c. tumors, reduced tumor weight and vascular density, were associated with a decrease in Ki-67 expression (cell proliferation), increases in TUNEL-positive lesions and expression of apoptotic proteins (caspase-3 (active)). The staining results for Ki-67, TUNEL and caspase-3 (active) are summarized in **Table** **3**.

## Discussion

We recently reported that CD44 and CD147 co-localize on both primary and metastatic CaP cells, and that over-expression of CD44v3-10 and CD147 is associated with CaP progression; Furthermore, we found a clear inverse relationship between DTX sensitivity and CD44v3-10/CD147 expression in CaP cell lines [21], [24]. In the present study we further investigated the roles of CD44 or CD147 in CaP metastasis and DTX responsiveness using *in vitro* CD44 or CD147-KD CaP cell lines and an *in vivo* s.c. xenograft model.

Previous reports have shown that CD44 and CD147 are close partners in various cancers [30]–[32]. CD147 stimulates production of HA [33], an extracellular polysaccharide that promotes tumor chemoresistance through interactions with the cell surface receptor CD44 [34]–[36]. CD147, through interactions with HA receptors (CD44) [36] and membrane-bound transporters [37], facilitates tumor cell chemoresistance. In addition, disruption of HA interactions with its cognate receptors interferes with CD147-mediated drug resistance [36] in part through disruption of protein complexes containing CD147 [30], [31]. In the present study, we found that knocking down either CD44 or CD147 affects the expression of the other protein to a lesser extent, as well as impacting on the expression of drug resistance protein (MRP2) and transporter protein (MCT4) in CaP cells. These data suggest that CD44 and CD147 are co-regulated and associated with MRP2 and MCT4 in CaP cells, and may play an important role in CaP metastasis and chemoresistance, although this remains to be confirmed in further studies.

The association of CD44 and CD147 with MCT transporter proteins has been documented for different cancers [30], [31], [37]–[39]. Metastatic cancer cells increase glucose consumption and metabolism via glycolysis, producing large quantities of lactate. Up-regulation of glycolysis and adaptation to acidosis are key events in the transition from *in situ* to invasive cancer [40]. The rapid transport of lactate by MCTs is of critical importance for almost all cells, especially tumor cells with elevated levels of glycolysis resulting in a decrease in extracellular pH. MCT4 and CD147 overexpression have been reported to be associated with poor prognosis in CaP [41]. Rudrabhatla *et* *al* reported that MCT4 is an important efflux pump for lactate and that the accumulation of lactate in the microenvironment may stimulate HA production and contribute to an acquired malignancy in melanoma cells [42]. MRP2 is one of 48 human ATP-binding cassette (ABC) transporters, also called ABCC2/the canalicular multiple organic anion transporter (cMOAT), and plays a role in the occurrence of the MDR phenotype in cancer cells [43]. An association with MCT4 or MRP2 is often an attribute of an aggressive phenotype and drug resistance in cancers [24], [44], [45]. CD147 silencing has been reported to inhibit MCT1/MCT4 and reduce the malignant potential of pancreatic cancer cells *in vivo* and *in vitro* [46]. The mechanisms underlying how CD44 and CD147 may regulate or influence MCT4 and MRP2 are not well defined. However, modifications of MCT4 and MRP2 can influence tumor growth and sensitivity to chemotherapy, and are a useful approach for cancer treatment [47], [48]. In the current study, we found that reducing CD44 or CD147 expression related to the concurrent reduction of MCT4 and MRP2 could increase the sensitivity of CaP cells to DTX treatment. This suggests that the effect of the DTX-related response after CD44 or CD147-KD in CaP cells may be functionally related to MCT4 and MRP2 expression and particular signaling pathways. In addition, CD44-KD clearly enhanced the greater sensitivity to DTX compared to CD147 KD cells, suggesting a possible stronger inhibition of survival pathways.

Colony formation assays provide a more appropriate measure of the long-term effects of potential therapeutic agents, assessing the ability of cells to retain proliferative potential after treatment, a characteristic that clinically facilitates tumor recurrences in patients. Our clonogenic assays indicated that downregulated expression of CD44 or CD147 suppressed the survival potential of CaP cells in the presence of either vehicle or DTX treatment. The sensitivity to DTX treatment was also higher in CD44 or CD147-KD cells compared to control cells, suggesting that CD44 and CD147 play an important role in CaP growth and DTX resistance. Wang *et* *al* reported that inhibition of CD147 expression reduces tumor cell invasion in PC-3 CaP cells via RNA interference [49]. Zhu *et* *al* also found that CD147 regulates cell adhesion, invasion, and cytoskeleton reorganization in PC-3 CaP cells using a shRNA approach [50]. In this study, we also found reduced *in vitro* invasion for CD44 or CD147-KD cells compared to control cells; this was greater in CD44-KD cells indicating that both CD44 and CD147 affect CaP invasion and that CD44 plays a more important role in invasion than CD147 does. The matrigel invasion assay mimics the ECM microenvironment by providing growth factors and creating a matrix scaffold for tumor cells to invade through. One possible mechanism for the reduced invasion observed in this study could be reduced levels of MMPs (for example, MMP-9) as observed with gelatin zymography for CaP cells with reduced CD44 or CD147 expression (unpublished observation).

PI3K/Akt and MAPK/Erk signaling pathways are related to CaP invasion [51], [52]. PI3K activation can induce chemoresistance in CaP cells through the up-regulation of MRP1 [53]. Activated forms of Akt can also increase the drug resistance of advanced CaP, and the PI3K/PTEN/Akt pathway appears to be more prominently involved in CaP drug resistance than the Raf/MEK/Erk pathway [54]. One recent study reported that PTEN/PI3K/Akt pathways are critical for maintaining a CaP stem-like cell (CD44^+^/CD133^+^) phenotype and that targeting PI3K signaling may be beneficial in CaP treatment by eliminating CaP stem-like cells [55]. We hypothesized that CD44 and CD147 might be involved in PI3K/Akt or MAPK/Erk signaling pathways in CaP metastasis and drug resistance. In the current study, we found that CD44 or CD147-KD could reduce both p-Akt and p-Erk levels in CaP cells. A more obvious reduction of p-Akt and p-Erk was seen in CD44-KD cells, suggesting that both PI3K/Akt and MAPK/Erk signaling pathways are involved in CD44/CD147-mediated CaP metastasis and drug resistance, and that CD44 plays a prominent role in this regulation compared to CD147. These findings are consistent with the colony assay, cell invasion and DTX responsiveness results. The role of CD44 and CD147 in the regulation of these two signaling pathways will be investigated in future studies.

To further validate our *in vitro* findings, we also studied an s.c. xenograft animal model. Metastases derived from s.c. human CaP PC-3 xenografts line have been reported [56] and we have previously described the development of CaP lymph node metastases in 100% of nude mice injected with PC-3 cells after 8 weeks [26]. The PC-3M-luc xenograft model we developed has 100% tumor take, with 100% local regional lymph node metastases post 8 weeks cell inoculation. This provides an appropriate model to mimic clinical CaP metastasis. Our findings indicated that CD44 or CD147-KD with VC treatment strongly regressed tumor progression for at least 8 weeks. Tumor volumes at the end of experiment in the CD44 or CD147-KD mice decreased by 37% and 35% respectively compared with the scr-controls. After 3 weeks continuous treatment with DTX, obvious tumor growth regression was found in CD44- or CD147-KD mice compared with the controls. The sensitivity rate to DTX treatment was higher in CD44- or CD147-KD cells compared to control cells. These results further confirmed the *in vitro* colony assay observations. CD44- or CD147-KD cell lines treated with VC or DTX can also completely prevent lymph node metastases for at least 8 weeks, during which period all control mice treated with VC developed lymph node metastases. These results suggest that CD44- or CD147-KD inhibit the growth of PC-3M-luc tumors, increase DTX response and prevent local regional lymph mode metastases. Furthermore, combining CD44- or CD147-KD with DTX could have advantages over a single treatment approach and can greatly reduce tumor growth and reduce tumor burden.

In the current study, we used shRNA lentiviral transfection for the CD44- or CD147-KD cell lines to stably reduce CD44 and CD147 expression and ensure continued downregulation during the *in vivo* tumorgenecity study. Our results indicate that *in vitro* CD44 or CD147-KD remained downregulated in the *in vivo* biological system for 8 weeks post-cell inoculation, and that the data from the current model system are reliable. We also found that compared to control cell lines, CD44- and CD147-KD affected the expression of each other in the animal xenografts, consistent with the *in vitro* findings from the same cell lines (**Fig.** **1**).

In CD44- or CD147-KD xenografts treated with VC or DTX, we found a comparatively dispersed tumor structure, with evidence of scattered targeted areas. Angiogenesis plays a critical role in tumor progression, providing adequate nutritional and oxygen supply essential for tumor proliferation and metastasis, and promoting tumor recovery following cytotoxicity [57]. In fact, the angiogenic potential of a tumor is directly correlated with poor prognosis [58]. In the present study, we found a profoundly reduced MVD (CD31 expression/hpf) in the CD44 or CD147-KD xenografts, especially the CD147 KD tumors, suggesting that CD44 and CD147 are related to CaP angiogenesis and that targeting CD44 and CD147 can inhibit CaP angiogenesis. CD147 is reported to affect angiogenesis mainly via three mechanisms: a) Inducing proteinases which cleave VEGF from the ECM; b) Stimulating the production of tumor-derived VEGF; and c) Increasing VEGFR2 expression on the endothelial cells [59]. However, the exact mechanism by which CD147 affects angiogenesis in CaP remains to be determined. As for how CD44 can influence CaP angiogenesis, one possibility is that CD44 regulated MMP production cleaves ECM-bound VEGF, similar to CD147. We have found reduced MMP-9 production in CD44-KD cells (unpublished data). Another possibility is that CD147-KD induced inhibition of angiogenesis leads to a secondary CD44-associated effect of tumor vasculature.

DTX is now considered the preferred chemotherapeutic agent for castrate-resistant prostate cancer (CRPC) and bone metastasis [60]. The most widely described mechanism by which DTX achieves this effect is through its activity as a mitotic spindle poison, disrupting microtubule dynamics and inducing G2/M cell cycle arrest, a downstream effect thought to be related to the phosphorylation of Bcl-2 [61]. As expected, DTX could inhibit tumor growth compared with VC treatment (*P*<0.05, see **Fig.** **3**), with larger targeted areas and reduced MVD in DTX-treated mice. In a recent study, we also demonstrated that DTX could inhibit epithelial ovarian cancer (EOC) growth in an i.p. OVCAR-3 model through inhibition of tumor angiogenesis [28]. In the current study, CD44 or CD147-KD xenografts treated with DTX, showed significant targeted areas with tumor islands and obviously reduced MVD, suggesting that DTX may have a similar anti-angiogenic function as seen in the EOC model [28]. The exact mechanisms by which the combination of CD44 or CD147-KD and DTX affect angiogenesis and vascular regression will be investigated in future studies.

As described above, CD44 or CD147-KD could effectively inhibit tumor growth and reduce tumor volume and MVD combined with DTX treatment. While the mechanisms for this remain unclear, we conducted preliminary studies to determine whether apoptosis was involved, including TUNEL and caspase-3 activation assays; caspase-3 activation is critical for the proteolytic cleavage of many key proteins during apoptosis [62]. We observed reduced cell proliferation in CD44 or CD147-KD and DTX-treated xenografts, which was further reduced in CD44/CD147-KD xenografts combined with DTX treatment. The number of apoptotic cells (TUNEL-positive) and caspase-3 (active) positive cells in PC-3M-luc-CD44/CD147-KD tumors also increased, consistent with tumor regression being related to reduced cancer cell proliferation and apoptosis.

In summary, we have demonstrated for the first time that both CD44 and CD147 are involved in CaP proliferation, invasion and chemoresistance, possibly mediated through activation of both PI3K/Akt and MAPK/Erk pathways *in vitro*. Furthermore, reducing either CD44 or CD147 expression effectively reduced CaP tumor growth, enhanced the response to DTX, reduced angiogenesis and induced apoptosis *in vivo*. Consequently, combination targeting of CD44 and CD147 with DTX provides a potent therapeutic approach for advanced, recurrent, CRPC.

## Supporting Information

Figure S1 **Lymph node metastases in different CaP groups after treatments.** Representative images demonstrating lymph node metastases (LNM) in histology (H&E staining) 8 weeks post cell inoculation in different CaP groups with different treatments. LNM was found in PC-3M-luc xenograft mice (**A**) and PC-3M-luc-scr xenograft mice (**B**) but not in PC-3M-luc-CD44-KD xenograft mice (**C**) and PC-3M-luc-CD147 xenograft mice (**D**). The arrows indicate PC-3M-luc metastatic cancer cells in local regional lymph nodes. Magnification ×100, 200, 400 in all images. KD: knock down; scr: scrambled shRNA control.
(TIFF)

Table S1 **Sequences of CD44 and CD147 shRNA lentiviral transduction particles.**
(DOC)
