# Supplementary figures and images for: In Vitro and In Vivo Prostate Cancer Metastasis and Chemoresistance Can Be Modulated by Expression of either CD44 or CD147
Source: PLoS One. 2012 Aug 3;7(8):e40716. doi: 10.1371/journal.pone.0040716 (PMC3411712; doi:10.1371/journal.pone.0040716)

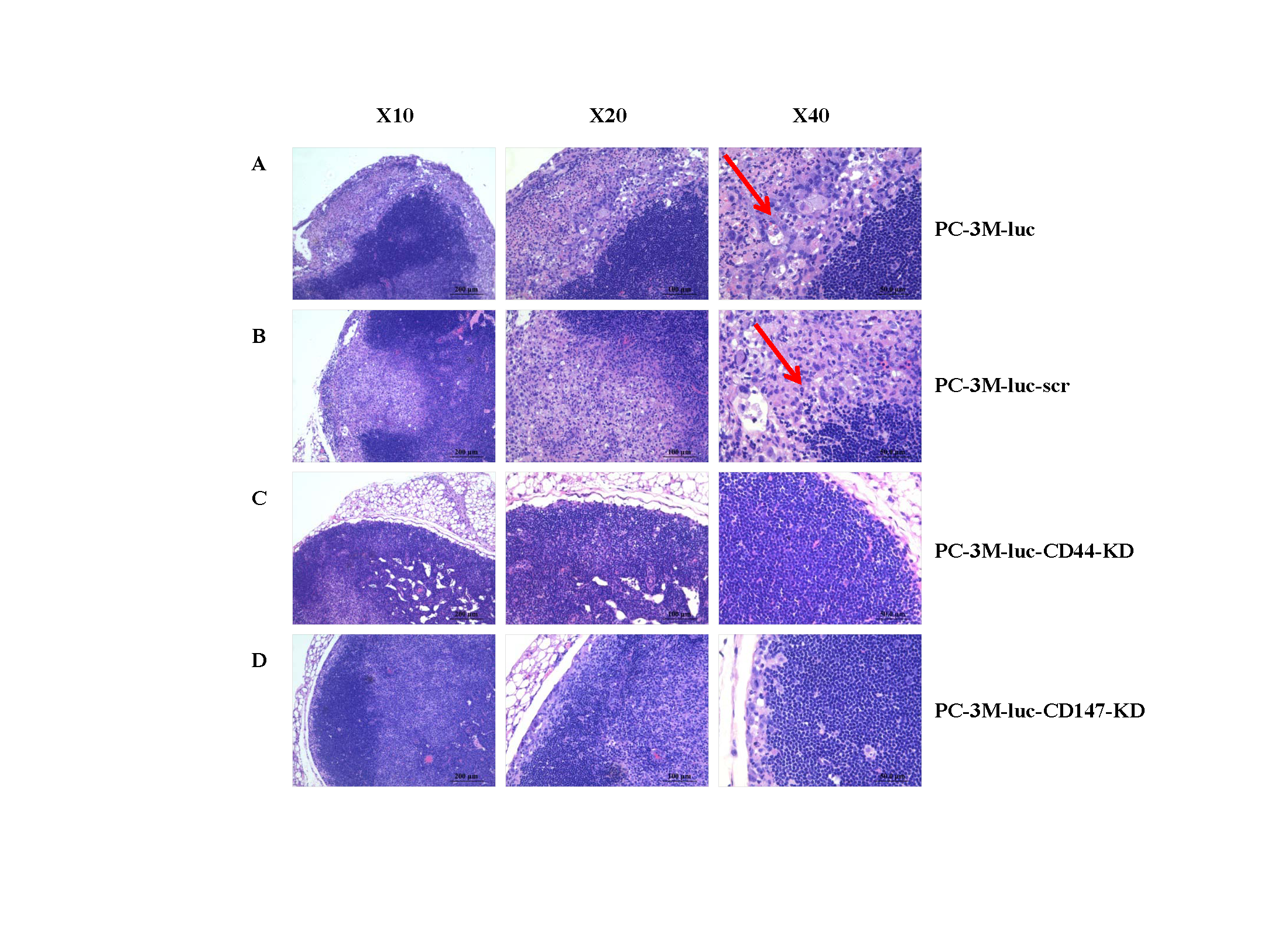

Supplement: Figure S1 — Lymph node metastases in different CaP groups after treatments. Representative images demonstrating lymph node metastases (LNM) in histology (H&E staining) 8 weeks post cell inoculation in different CaP groups with different treatments. LNM was found in PC-3M-luc xenograft mice (A) and PC-3M-luc-scr xenograft mice (B) but not in PC-3M-luc-CD44-KD xenograft mice (C) and PC-3M-luc-CD147 xenograft mice (D). The arrows indicate PC-3M-luc metastatic cancer cells in local regional lymph nodes. Magnification ×100, 200, 400 in all images. KD: knock down; scr: scrambled shRNA control. (TIFF) [file pone.0040716.s001.tif]
