# Supplementary material for: In Vitro and In Vivo Prostate Cancer Metastasis and Chemoresistance Can Be Modulated by Expression of either CD44 or CD147
Source: PLoS One. 2012 Aug 3;7(8):e40716. doi: 10.1371/journal.pone.0040716 (PMC3411712; doi:10.1371/journal.pone.0040716)
Supplement: Table S1 — Sequences of CD44 and CD147 shRNA lentiviral transduction particles. (DOC) [file pone.0040716.s002.doc]

**Table S1.** Sequences of CD44 and CD147 shRNA lentiviral transduction particles

| **CD44 shRNA lentiviral transduction particles** | A | CCGGCCAACTCTAATGTCAATCGTTCTCGAGAACGATTGACATTAGAGTTGGTTTTTG |
| --- | --- | --- |
|  | B | CCGGGCCCTATTAGTGATTTCCAAACTCGAGTTTGGAAATCACTAATAGGGCTTTTTG |
|  | C | CCGGCCTCCCAGTATGACACATATTCTCGAGAATATGTGTCATACTGGGAGGTTTTTG |
|  | D | CCGGCGCTATGTCCAGAAAGGAGAACTCGAGTTCTCCTTTCTGGACATAGCGTTTTTG |
|  | E | CCGGCGGAAGTGCTACTTCAGACAACTCGAGTTGTCTGAAGTAGCACTTCCGTTTTTG |
| **CD147 shRNA lentiviral transduction particles** | A | CCGGCCAGAATGACAAAGGCAAGAACTCGAGTTCTTGCCTTTGTCATTCTGGTTTTT |
|  | B | CCGGCCCATCATACACTTCCTTCTTCTCGAGAAGAAGGAAGTGTATGATGGGTTTTT |
|  | C | CCGGGCTACACATTGAGAACCTGAACTCGAGTTCAGGTTCTCAATGTGTAGCTTTTT |
|  | D | CCGGACAGTCTTCACTACCGTAGAACTCGAGTTCTACGGTAGTGAAGACTGTTTTTT |
|  | E | CCGGGAAGTCGTCAGAACACATCAACTCGAGTTGATGTGTTCTGACGACTTCTTTTT |

**Note:** shRNA: Short hairpin RNA
